# MRI guided breast biopsy: initial experience of service expansion in West Midlands

**DOI:** 10.1186/bcr3774

**Published:** 2015-11-05

**Authors:** Muthyala Sreenivas, Vandana Gaur

**Affiliations:** 1 UHCW NHS Trust, Coventry, UK

## Introduction

At UHCW Hospitals NHS Trust (which is the sole provider of diagnostic breast MRI for UHCW, South Warwickshire and George Elliot Hospitals NHS Trusts) the MRI guided breast biopsy service has been running since June 2011. Initially, the service was offered to patients imaged at UHCW NHS Trust. With increased experience and confidence the service is now offered to all the eight NHSBSP screening sites in the West Midlands. Here we present our experience regarding the outcomes.

## Methods

Since June 2011, 50 cases were referred for MRI guided breast biopsy of which 10 cases were referred from outside the UHCW NHS Trust diagnostic imaging cohort. There were three high-risk screening cases whilst the remaining cases already had diagnosis of cancer but MRI identified further lesions. All cases were graded B3 or above on diagnostic imaging. All images were reviewed (obtained via IEP) and biopsy performed within 10 days of the initial request. Biopsy samples were sent to local hospitals for pathological analysis.

## Results

Forty-eight out of 50 cases were considered for biopsy. Two cases were deemed benign (one on review of diagnostic MRI and one case on second-look US). There were 24 malignancies (50 % of all cases). A follow-up MRI or surgical excision recommendation was made as necessary for non-malignant cases in the biopsy report.

## Conclusion

MRI guided breast biopsy has been successfully established at UHCW NHS Trust which currently serves as the regional referral centre in the West Midlands.

